# The Present Status of Suspension in the Treatment of Locomotor Ataxia

**Published:** 1890-06

**Authors:** 


### THE PRESENT STATUS OF SUSPENSION IN THE TREATMENT OF LOCOMOTOR ATAXIA.

In the *Progrès Médical* of April 26, 1890, Dr. Raoult, of Paris, examines the results obtained by different observers, in different countries, who have conscientiously used this mode of treatment for ataxia. It seems that in England and America the results have not been as favorable as those obtained in Germany and France. An American observer explains this by saying that the French people are more impressionable than the Anglo-Saxons, and present symptoms more functional than organic. In all probability said observer has been looking for a cure instead of amelioration.

Among the recent contributions to this subject the majority still favor suspension. Balaban, of Paris, in his Thèse de Paris, reports nine cases of tabes treated in this manner under the direction of Dujardin-Beaumetz. In eight cases the gait was considerably improved, and the incoördination and lightning pains diminished. In one case the vesical troubles disappeared, and in three cases the anesthesia. In but one case were the results unsatisfactory, and treatment was suspended after twenty-five trials.

Dujardin-Beaumetz reports twenty-five cases treated at L'Hôpital Cochin, with good results in the majority of cases. In four patients the gait was much improved, while in six patients no improvement was noticed. In those cases where the improvement was but slight, it occurred before the fifteenth day of treatment. After that time the symptoms remained stationary.

Dr. Ladame treated fifteen cases of locomotor ataxia, twelve males and three females. He reports on eleven cases, four having disappeared. Of eleven cases only two were not benefited by suspension.

Dr. Mouisset treated eight atactics, and in one case only was the method unavailing.

Dr. Thiberhein reports twenty-six cases, nineteen of whom took ten or more treatments. Of these, seventeen have been ameliorated and two not.

Observers in Germany and Russia have likewise met with favorable results in the majority of cases treated a sufficient length of time.

